# Salidroside Improves Antibiotic-Induced Gut Microbiota Disturbance and Low Levels of Short-Chain Fatty Acids in Mice

**DOI:** 10.3390/foods11193073

**Published:** 2022-10-03

**Authors:** Tong Sun, Jingyi Yang, Lili Lu

**Affiliations:** School of Pharmacy, Tongji Medical College, Huazhong University of Science and Technology, Wuhan 430030, China

**Keywords:** salidroside, antibiotics, gut microbiota, short-chain fatty acids, prebiotics

## Abstract

Salidroside is the main active constituent of the functional food *Rhodiola rosea* and has a wide range of biological activities. This work investigated the regulation of salidroside with different doses and durations on the gut microbiota disturbance resulting from excessive injection of antibiotics in mice C57BL/6J. The salidroside treatment attenuated antibiotic-induced intestinal damage and reduced the levels of inflammation factors such as TNFα and IL-6. Importantly, salidroside promoted the recovery of gut microbiota richness, diversity, and community structure in mice. The intake of salidroside increased the abundance of beneficial bacteria represented by *Lactobacillus* and *Bifidobacterium*, and reduced the portion of disease-related bacteria, thus reshaping the structure and composition of gut microbiota. Moreover, after ingesting salidroside, the contents of short-chain fatty acids (SCFAs) increased, which could also facilitate the recovery of intestinal barrier functions. Low-dose salidroside plays an especially superiorly beneficial role in promoting the proliferation of probiotics and the production of SCFAs in a short time. These findings suggested that salidroside had similar beneficial functions of prebiotics and thus intake of it might be a new promising food therapy for improving antibiotic-induced gut microbiota disturbance.

## 1. Introduction

Gut microbiota plays an important role in the physiological and pathological processes of human body. It helps extract additional energy from food, regulates the intestinal endocrine function, drives intestinal development by thickening villus, resists against colonization by exogenous microorganisms, and directly inhibits the expansion of potential pathogens [1,2,3]. Gut microbiota can also influence nerve signaling, produce vitamins and secondary bile acids, and modify or influence specific drugs after intake of them [3]. On the other hand, an imbalance of the normal gut microbiota is associated with the development of metabolic disorders and even chronic diseases, including gastrointestinal, neurological, respiratory, hepatic, and cardiovascular diseases [3,4].

Antibiotics have been developed as powerful weapons to fight against pathogen infections. However, the continuous and excessive use of them brings seriously adverse effects, particularly exemplified by the broad-spectrum antibiotics that kill the beneficial host intestinal flora together with the pathogens, thus leading to gut dysbiosis in the structure and composition of the commensal microbiota along with the increased susceptibility to infections [2,5]. The effects of antibiotic abuse, such as the alteration of the microbial composition, are long-lasting, which may increase the risk of secondary infections, allergies, obesity, *Clostridioides difficile* infection, and the development of drug-resistant flora [6]. In order to maintain the micro-ecological balance of the gut microbiota, it is necessary to avoid non-essential use of antibiotics or repair antibiotic-induced gut microbiota disturbance by functional food or therapeutic agents. Recently, a variety of food supplements from natural products have been found to have the ability to adjust the intestinal microbiota in mice [7,8,9,10]. For example, the ginsenoside Rk3 can repair antibiotic-induced gut microbiota dysbiosis in C57BL6/J mice and improve colonic inflammation [11].

*Rhodiola rosea*, a worldwide botanical adaptogen, is known as the “golden root” or “roseroot” with various health-promoting effects [12]. It is a high-value functional and medicinal plant, commonly used as a “food supplement” for healthcare in China and other Asian countries, and also registered in the United Kingdom and throughout Europe with a traditional history as a commercially dietary supplement for the treatment of stress-induced fatigue, exhaustion, and so on [13,14]. The extract of *Rhodiola rosea* can alleviate dextran sulfate sodium-induced colitis, reduce the levels of inflammatory factors, mediate gut barrier integrity, and reshape the gut microbiota [15]. Salidroside [2-(4-hydroxyphenyl) ethyl-β-D-glucopyranoside], as the main component of *Rhodiola rosea* extract, exhibits significant bioactivities, such as neuroprotective, cardiovascular protective, immunomodulatory, anti-aging, anti-fatigue, anti-apoptotic, antioxidant, and anti-hypoxic effects [16]. More interestingly, salidroside has been discovered to alleviate certain diseases by regulating gut microbiota, such as furan-induced liver injury [17], high-fat diet-induced non-alcoholic steatohepatitis [18], and cognitive dysfunction [19]. Nevertheless, the effects of salidroside on the gut microbiota disturbance induced by antibiotics have not yet been reported.

Ceftriaxone sodium, a broad-spectrum cephalosporin belonging to the β-lactam class, is currently among the first-line therapeutic agents against infections, owing to its broad-spectrum antibacterial activity, clinical efficacy, and safety profile [20]. According to a survey including 23,572 patients from 56 countries, ceftriaxone was the most commonly prescribed antibiotic to hospitalized children in Africa, the Eastern Mediterranean, Europe, and Southeast Asia [21]. After intake, the ceftriaxone sodium is excreted mainly through the urine and partly through the bile, followed by entry into intestine and excretion with feces [6]. Both healthy human and patients treated by ceftriaxone led to gut microbiota community changes compared with non-treated ones, including the apparent loss of some species and lower diversity, which may lead to decreased resilience in gut ecosystems [22,23]. Moreover, some pathogenic bacteria were enriched in response to ceftriaxone, especially *Clostridium difficile* [22]. It was reported that the extended-spectrum β-lactamase-producing *Enterococcus* occurred and persistently existed in the gut microbiota of over a quarter of hospitalized patients treated with ceftriaxone [24]. 

The above problems can lead to immunosuppression and poor performance status [24]. Due to the widespread use and current problems of ceftriaxone sodium, a gut microbiota disturbance model was built based on the excessive use of this antibiotic, and the salidroside was first found to have the ability to repair the gut microbiota disturbance by promoting beneficial bacteria abundances and short-chain fatty acid (SCFA) production.

## 2. Materials and Methods

### 2.1. Animals and Materials

Specific pathogen-free (SPF) C57BL/6J mice (male, 7 weeks old, all weighing between 21–25 g) were purchased from SPF (Beijing) Biotechnology Co., Ltd. (Beijing, China). Ceftriaxone sodium was purchased from Shanghai Roche Pharmaceutical Ltd. (Shanghai, China). Salidroside was purchased from Solarbio Science & Technology Co., Ltd. (Beijing, China). All other chemicals and reagents were of analytical grade.

### 2.2. Experimental Design

Models of gut microbiota disturbance were built as follows. After one week of adaptive feeding, 20 mice were randomly and equally divided into 4 groups (*n* = 5 for each group): Con group (0.9%/day NaCl), Low group (200 mg/kg/day ceftriaxone sodium), Medium group (400 mg/kg/day ceftriaxone sodium), and High group (800 mg/kg/day ceftriaxone sodium). The saline or ceftriaxone sodium were taken by intraperitoneal injection. The experiments were conducted for a total of 3 days.

Salidroside repair tests were performed as follows. After one week of adaptive feeding, 30 mice were randomly divided into 6 groups (*n* = 5 for each group), including Con group (0.9% NaCl + 0.9% NaCl), Mod group (ceftriaxone sodium + 0.9% NaCl), Sal25 group (ceftriaxone sodium + 25 mg/kg/day salidroside), Sal50 group (ceftriaxone sodium + 50 mg/kg/day salidroside), Sal100 group (ceftriaxone sodium + 100 mg/kg/day salidroside), and Sal200 group (ceftriaxone sodium + 200 mg/kg/day salidroside). The Con group was injected intraperitoneally with saline, while the other groups were injected intraperitoneally with 400 mg/kg/day ceftriaxone sodium for 3 days. Then the Con and Mod groups were gavaged with saline, and the Sal groups involving Sal25, Sal50, Sal100, and Sal200 were gavaged with salidroside at different concentrations for 2 weeks.

During the experimental period, the mice were fed and watered freely in an SPF environment under controlled conditions including temperature of 22 ± 2 °C, relative humidity of 50 ± 10%, and 12/12 h light/dark cycles, and the bedding was changed 2–3 times a week. All experiments were approved by the Animal Care and Use Committee of Huazhong University of Science and Technology (IACUC Number: 2642).

### 2.3. Sample Collection, Biochemical Parameter Analysis, and Histological Analysis

The body weight and activity status of the mice were recorded daily. After treatment, fresh feces of mice were collected and immediately stored in liquid nitrogen and then kept at −80 °C. The blood was centrifuged at 1000 *g* for 20 min to prepare the serum. The levels of alanine aminotransferase (ALT) and aspartate aminotransferase (AST) were tested under the instruction of corresponding assay kits (Nanjing Jiancheng Bioengineering Institute, Nanjing, China), and the levels of TNFα and IL-6 were tested by corresponding ELISA kits (Boster Biological Technology Co., Ltd., Wuhan, China).

After taking blood samples, the mice were sacrificed by decapitation and dissected immediately to obtain their spleens, ilea, and colons. The spleen was weighed to calculate the spleen index. Spleen index = spleen weight (g)/mouse body weight (g) × 100.

The ileum and colon samples were fixed in 4% paraformaldehyde for 24 h under room temperature, followed by embedding in paraffin, sectioning, and staining with hematoxylin and eosin dye. The tissue slices were observed under an inverted microscope (OLYMPUS, Tokyo, Japan).

### 2.4. DNA Extraction and 16S rRNA Sequencing

Total DNA of fecal microorganisms was extracted using the E.Z.N.A.^®^ soil DNA Kit (Omega Bio-tek, Norcross, Georgia, USA). The hypervariable regions V3–V4 of the bacterial 16S rRNA gene were amplified with primers 338F (5′-ACTCCTACGGGAGGCAGCAG-3′) and 806R (5′-GGACTACHVGGGTWTCTAAT-3′) by a PCR thermocycler (ABI GeneAmp^®^ 9700, Foster City, California, USA). The PCR products were extracted and purified by agarose gel. Purified amplicons were sequenced by the Illumina Miseq PE300 platform (Illumina, San Diego, California, USA). Raw 16S rRNA gene sequences were quality-filtered based on fastp software and merged by FLASH software.

### 2.5. Analysis of Gut Microbiota

Operational taxonomic units (OTUs) with a 97% similarity cutoff were clustered using UPARSE software, and then chimeric sequences were identified and removed. The taxonomy of each OTU representative sequence was analyzed by RDP Classifier against the 16S rRNA database (silva138/16s) using the confidence threshold of 0.7.

### 2.6. Extraction and Analysis of SCFAs

Fecal samples were ground twice and sonicated for 10 min in 0.5% phosphoric acid solution. The resulting suspension was centrifuged, and the supernatant was extracted with *n*-butanol solvent and centrifuged again. The resulting supernatant was analyzed by gas chromatography, which was performed through Agilent 8890B gas chromatography coupled with Agilent 5977B mass selective detector containing an inert electron impact ionization source (Agilent Technologies Inc., Santa Clara, CA, USA). The ionization voltage was 70 eV.

Chromatographic conditions were as follows. The samples were separated by an HP-FFAP capillary column (30 m × 0.25 mm × 0.25 µm), with 99.999% helium as a carrier gas at a constant flow rate of 1 mL/min. The injection volume of samples was 1 µL, which was introduced in splitting mode (10:1) with the inlet temperature at 260 °C. The ion source temperature was 230 °C and the quadrupole temperature was 150 °C. The GC column temperature was programmed to hold at 80 °C, rose to 120 °C at a rate of 40 °C /min, then rose to 200 °C at a rate of 10 °C /min, and finally held at 230 °C for 6 min. Data acquisition was conducted on full scan mode with a range of *m/z* 30–300.

### 2.7. Statistical Analysis

All data were displayed as the mean ± standard error of the mean (SEM). The statistical significance between the two groups was analyzed by Student’s *t*-test. Among three or more groups, when the data is normally distributed and variances were homogeneous, the statistical significance was analyzed by one-way ANOVA test, or else the Welch ANOVA test was applied. *p* < 0.05 was considered to be statistically significant.

## 3. Results

### 3.1. Effects of Excessive Ceftriaxone Sodium on Mice and Their Gut Microbiota

The model of gut microbiota disturbance was built by intraperitoneal injection of excessive ceftriaxone sodium at 200–800 mg/kg/day. The proper dosage of ceftriaxone sodium was preliminarily determined based on the extent of damage to the spleens, intestine tissues, and the levels of inflammation factors of mice (Figure S1). Regardless of the ceftriaxone sodium dose, no apparent changes were observed in the body weight. However, there was a significant decrease in the spleen index of mice in all antibiotic-treated groups (*p* < 0.001), suggesting a decline in the immune function of mice. After antibiotic treatment, biochemical indicators of liver injury such as ALT and AST, as well as inflammatory cytokines such as TNF-α and IL-6, were all increased in Medium groups compared with the Con group (*p* < 0.05), indicating 400 mg/kg/day ceftriaxone sodium caused liver injury and inflammatory response in mice.

Intestinal barrier was seriously damaged based on the results of histological analysis. The changes of gut microbiota in mice after antibiotic treatment were further detected by 16S rRNA high-throughput sequencing. The results showed that 400 mg/kg/day ceftriaxone sodium induced obvious alteration to the gut microbiota, with the diversity and richness of the gut microbiota reduced.

#### 3.1.1. Effects of Excessive Ceftriaxone Sodium on Mice Intestine

Excessive ceftriaxone sodium at 200–800 mg/kg/day resulted in an obvious change in the morphology of the mice cecum, with the characteristics of significant edema and darkened color, whereas the Con group without antibiotic treatment had a normal cecum morphology with a bright yellow color (Figure S2). The alteration in the cecum morphology was presumably to result from intestinal inflammation. Additionally, ceftriaxone sodium caused varying degrees of damage to the ileum and colon (Figure S2). In the ileum, the muscular layer of the intestinal wall had thinned and the goblet cells were lost, especially in the Medium and High groups. In the colonic region, the intestinal villi in the Low and Medium groups were less tightly attached to the muscular layer, and the gap between the intestinal villi and muscular layer in these two groups became larger than in the Con group. These results, combined with the data of inflammatory cytokines mentioned above, showed that the amount of 400 mg/kg/day ceftriaxone sodium used in the Medium group was sufficient to cause obvious intestinal damage and inflammation, and thus the changes in the gut microbiota of the Medium group were further investigated.

#### 3.1.2. Effects of Excessive Ceftriaxone Sodium on Gut Microbiota in Mice

The 16S rRNA of the gut microbiota from the Con and the Medium groups were sequenced. In comparison with the Con group without antibiotic treatment, the Medium group treated with 400 mg/kg/day ceftriaxone sodium had serious destruction in the gut microbiota. The community richness and diversity indexes and the composition at both phylum and genus levels dropped dramatically.

The Chao index that reflects the community richness and the Shannon index that responds to the diversity of the community were selected to evaluate the α-diversity (Figure S3). The Chao and Shannon indexes in the Medium group were significantly lower than those in the Con group (*p* < 0.01), indicating the richness and diversity of the gut microbiota were reduced. Principal co-ordinates analysis (PCoA) based on Bray–Curtis distance showed a separation in the gut microbiota structure at the OTU level between Con and Medium groups (Figure S3). According to the Veen analysis in Figure S3, the two groups shared 43 common OTUs. The Con group had 485 unique OTUs, whereas the Medium group had only 11 unique OTUs. These results fully confirmed that the gut microbiota similarity between the Con and Medium groups was highly reduced.

The composition that can be detected at both phylum and genus levels decreased after ingestion of ceftriaxone sodium. At the phylum level, the abundance of *Firmicutes* was increased, whereas the abundance of *Bacteroidota* was decreased. Thus, the F/B ratio in Medium group (494.76%) was much higher than Con group (125.88%). Additionally, the phyla of *Verrucomicrobiota* and *Actinobacteria* disappeared. When analyzing at the genus level, the dominant fecal microflora *norank_f_Muribaculacea* and *Allobaculum* in the normal mice were almost undetectable in the antibiotic-treated ones. By contrast, there was an obvious increase in the abundances of *Parabacteroides* and *Enterococcus* after antibiotic treatment.

### 3.2. Effects of Salidroside on Antibiotic-Treated Mice and Their Gut Microbiota

**Figure 1:**
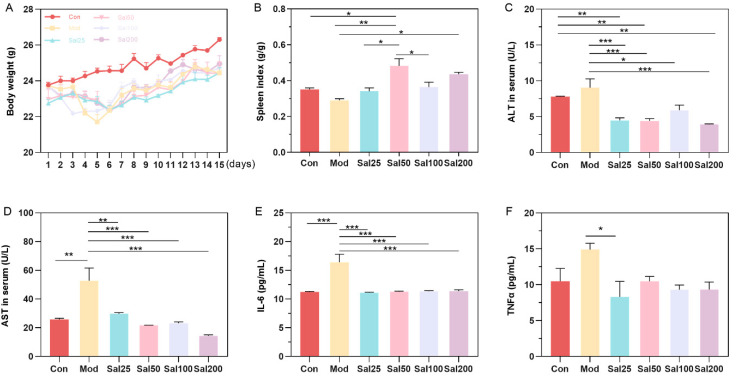
The effects of salidroside on mice weight (**A**), spleen index (**B**), and serum concentrations of alanine aminotransferase (ALT) (**C**), aspartate aminotransferase (AST) (**D**), IL-6 (**E**), and TNFα (**F**). The values are displayed as mean ± SEM. * *p* < 0.05, ** *p* < 0.01, *** *p* < 0.001.

After intraperitoneal injection of 400 mg/kg/day ceftriaxone sodium for three days, mice were gavaged with different doses of salidroside or 0.9% NaCl. Mice in Mod and all Sal groups administered with ceftriaxone sodium showed a slight decrease in body weight compared to the Con group within 5 days (Figure 1). After 5 days, the mice in each group gained body weight evenly without apparent differences. The intake of salidroside raised the spleen index of mice in all Sal groups compared to the Mod group, among which the spleen index of the Sal50 group was increased most significantly (*p* < 0.01) (Figure 1). ALT and AST, as biochemical indicators of liver injury, were all decreased after salidroside treatment for 7 days (*p* < 0.05), indicating the improvement of liver injury. Essential inflammatory cytokines such as TNF-α and IL-6 were also decreased in all Sal groups, suggesting the alleviation of inflammation.

Whether the salidroside had a repairing effect on gut microbiota disturbance was further investigated. Fresh feces of mice were taken on the 7 and 14th days, respectively, after administration with salidroside. The results showed that salidroside not only promoted the repair of antibiotic-induced organ damage but also accelerated the recovery of gut microbiota.

#### 3.2.1. Salidroside Promoted the Recovery of Antibiotic-Induced Intestinal Damage

**Figure 2:**
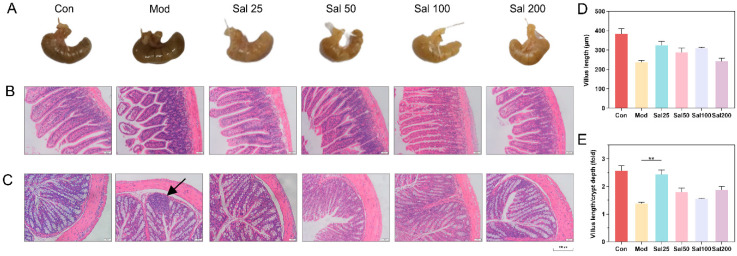
The effects of salidroside on mice intestinal damage. (**A**) Cecum tissues. (**B**) Ileum tissues. (**C**) Colon tissues. Scale bars indicate 150 μm. The black arrow indicates inflammatory infiltration. Histological analysis of the villus length (**D**) and the ratio of villus length to crypt depth (**E**).

The structural damage of the intestine induced by 400 mg/kg/day ceftriaxone sodium was improved in all groups administered with salidroside for 7 days (Figure 2). The color and morphology of the cecum in all Sal groups recovered to be nearly similar to the Con group without antibiotic treatment, whereas the cecum in the Mod group administered with the antibiotic but without salidroside still remained black as before. The ileal structure in the Sal groups tended to be normal, whereas the ileal villi of the Mod group remained damaged and detached. Histological analysis further showed the length of villus and the ratio of villus length to crypt depth in Sal groups were higher than those in the Mod group. In terms of colonic structures, the Mod group had inflammatory infiltration in the colon on the 7th day. By contrast, the Sal groups exhibited reduced colonic mucosal edema without inflammatory infiltration. The colonic villi of the groups Sal50, Sal100, and Sal200 were more tightly attached to the muscular layer than the Sal25 and Mod groups. All the results proved that salidroside had an ameliorative effect on intestinal damage and inflammation induced by ceftriaxone sodium.

#### 3.2.2. Salidroside Modulated Antibiotic-Induced Gut Microbiota Disturbance

**Figure 3:**
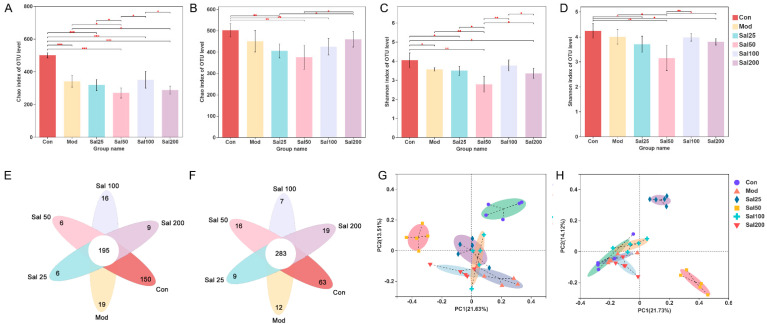
The effects of salidroside on the structure of gut microbiota. (**A**,**B**) Changes in Chao indexes after 7 and 14 days, respectively. (**C**,**D**) Changes in Shannon indexes after 7 and 14 days, respectively. (**E**,**F**) Veen analysis after 7 and 14 days, respectively. (**G**,**H**) Principal co-ordinates analysis (PCoA) analysis after 7 and 14 days, respectively. * *p* < 0.05, ** *p* < 0.01, *** *p* < 0.001.

Effects of salidroside on the regulation of gut microbiota disturbance induced by ceftriaxone sodium were further investigated. As shown in Figure 3, the Chao index of the Sal100 group administered with salidroside for 7 days was higher than that of the Mod group but did not reach the level of the Con group, suggesting the community richness of gut microbiota was not fully restored. After 14 days, the Chao index of Sal200 almost recovered to the level of the Con group. As for the Shannon index, the values were slightly lower in all Sal groups except for Sal100 than that in the Mod group on the 7th day. No significant differences were observed between the groups of Sal100 and Con, indicating that these two groups were similar in community diversity on the 7th day. The same trend was maintained on the 14th day. Whether on day 7 or day 14, the Sal25 and Sal50 groups always had slightly lower Chao and Shannon indexes than other groups, indicating lower richness and diversity. The results of the PCoA analysis showed that on the 7th day, the distributions of Mod, Sal25, Sal100, and Sal200 groups were close to each other, indicating their similarity in community structure. On the 14th day, the groups of Con, Mod, Sal100, and Sal200 displayed close distributions, with the Sal25 and Sal50 groups different from them. The compositional similarity and overlap of each group were analyzed at the OTU level by Veen plot. In comparison with the results on the 7th day, the number of total common OTUs in all groups increased after a 14-day salidroside treatment, indicating the gut microbiota composition among different groups became more similar with extended treatment time. The intake of salidroside increased the similarity of the overall gut microbiota composition between the Con and Sal groups, indicating that salidroside improved the structure recovery of gut microbiota. It should be noted that except for the Sal100 group, the number of unique OTUs of the Sal groups increased, suggesting that salidroside might promote the emergence of some distinct microbiota.

**Figure 4:**
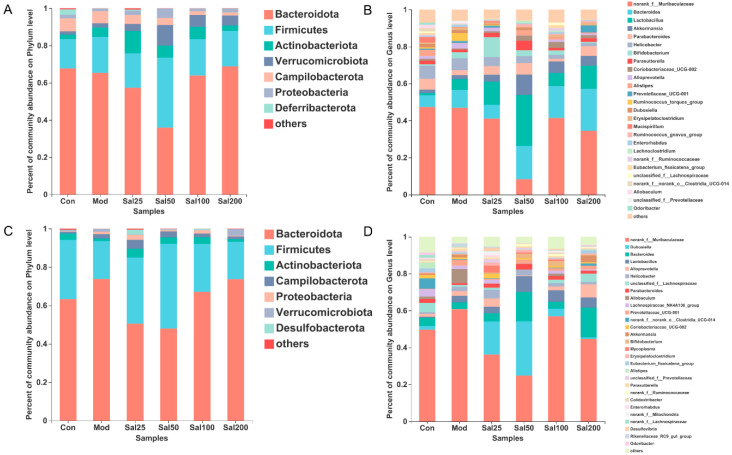
The effects of salidroside on the gut microbiota composition. (**A**,**C**) The composition at the phylum level after 7 and 14 days, respectively. (**B**,**D**) The composition at the genus level after 7 and 14 days, respectively.

At the phylum level, *Bacteroidota* and *Firmicutes* still dominated after salidroside treatment for 7 and 14 days (Figure 4). On the 7th day, the *Firmicutes*/*Bacteroidota* ratio of the Sal25 (32.44%), Sal50 (105.05%), and Sal100 (30.56%) groups were higher than that of the Mod (29.65%) and Con (23.15%) groups, among which the Sal50 group showed the largest ratio. The abundances of *Actinobacteria* phylum in the groups of Sal25 (11.95%), Sal50 (6.54%), and Sal100 (6.64%) were strikingly higher than those in the Mod (5.10%) and Con (2.41%) groups on the 7th day, the trend of which maintained until the 14th day. In particular, the increase of *Actinobacteria* in the Sal25 group was the most significant, indicating that low-dose salidroside could rapidly raise the proportion of *Actinobacteria*. Additionally, salidroside showed a significant promoting effect on the growth of *Verrucomicrobiota* on the 7th day. The abundances of *Verrucomicrobiota* in the Sal25 (3.76%), Sal50 (11.00%), Sal100 (6.27%), and Sal200 (5.37%) groups were higher than those in the Mod (2.12%) and Con (1.81%) groups.

At the genus level, salidroside also promoted the recovery of gut microbiota and even enhanced the abundances of certain beneficial bacterial populations (Figure 4). The abundances of *Bacteroides* in the groups of Sal50 (18.06%), Sal100 (17.20%), and Sal200 (22.72%) were higher than the Con (6.44%) and Mod (9.72%) groups after a 7-day treatment with salidroside. After 14 days, the abundances of *Bacteroides* in the Sal50 (15.86%) and Sal200 (16.10%) groups were still considerably higher than the Con (4.56%) and Mod (3.54%) groups. Additionally, the abundances of *Parabacteroides* in the groups of Sal25 (4.65%), Sal50 (6.17%), and Sal200 (5.17%) were higher than the Mod group (2.64%) after 7 days of treatment, among which the abundance of Sal50 even exceeded that in the Con group (5.92%). After 14 days, *Parabacteroides* still had higher proliferation in Sal groups (1.03–3.06%), with the abundances in Sal25 (2.29%) and Sal50 (3.06%) groups slightly higher than the Con group (2.02%). The abundances of *Dubosiella* in all Sal groups (0.87–29.48%) were higher compared to the Mod group (0.10%). The lower dose of salidroside in the Sal25 (18.00%) and Sal50 (29.48%) groups promoted the proliferation of *Dubosiella*, whereas the higher dose of salidroside exerted weak effects on the recovery of *Dubosiella*.

Along with the promotion of bacterial proliferation, salidroside also exhibited inhibitory effects on the growth of some bacteria at the genus level. The abundances of *norank_f_Muribaculaceae* in all Sal groups (8.30–41.43%) treated with salidroside for 7 days were lower than those in the Con (47.35%) and Mod (46.90%) groups, among which the largest inhibition of the salidroside on bacterial growth was observed in the Sal50 group (8.30%). On the 14th day, the abundances of this bacteria in the Sal25 (36.15%) and Sal50 (24.80%) groups were still lower than those in the Mod (60.64%) and Con (49.63%) groups. The salidroside-induced inhibitory effect on *norank_f_Muribaculaceae* in the Sal50 group was the most significant whether on the 7th day or the 14th day. *Helicobacter* abundance was also lower in the Sal groups (1.59–4.87%) than those in the Con (6.96%) and Mod (6.52%) groups on the 7th day. The inhibitory effect of salidroside on the growth of *Helicobacter* became more obvious with the increase of salidroside concentrations. Additionally, the levels of *Ruminococcus_torques_group* were lower in the Sal groups (0.00–0.54%) than those in the Con (0.74%) and Mod (4.26%) groups on the 7th day. Salidroside reduced the abundance of *Ruminococcus_torques_group* which had been proliferated after the treatment with ceftriaxone sodium.

**Figure 5:**
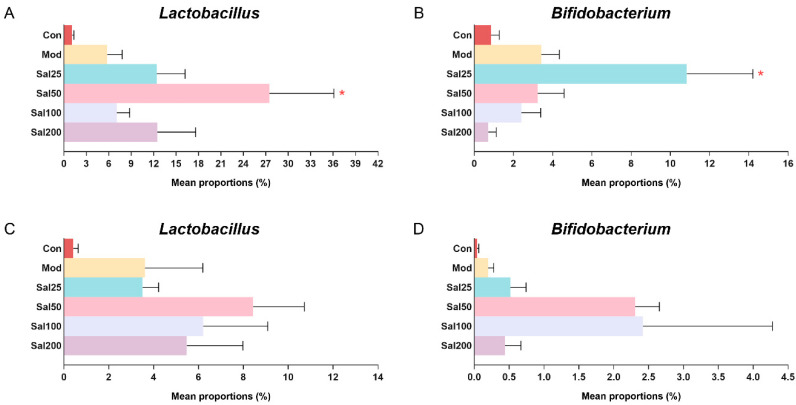
The effects of salidroside on probiotic abundance. (**A**,**C**) The abundance of *Lactobacillus* after 7 and 14 days, respectively. (**B**,**D**) The abundance of *Bifidobacterium* after 7 and 14 days, respectively. * *p* < 0.05 compared with the Mod group.

Due to the importance of probiotics in gut microbiota, special attention was focused on the effects of salidroside on the abundances of *Lactobacillus* and *Bifidobacterium* (Figure 5). It is well known that *Lactobacillus* has been used worldwide as a probiotic to exert extensive anti-inflammatory effect and against *Clostridium difficile* [25,26] and that *Bifidobacterium* contributes to host defense responses and prevention of infectious diseases [27]. The ingestion of salidroside was observed to stimulate the proliferation of probiotics in the mice. The abundances of *Lactobacillus* in the Sal groups (7.09–27.51%) after a 7-day treatment with salidroside were higher than the Con (1.11%) and Mod (5.80%) groups, which confirmed the contribution of salidroside to the growth of *Lactobacillus*. The most obvious increase in bacterial growth was observed in the Sal50 group whether it was on the 7th day or the 14th day. The abundance of *Bifidobacterium* in the Sal25 group (10.84%) was significantly higher than those in the Con (0.85%) and Mod (3.42%) groups after a 7-day salidroside treatment. When extending the treatment to 14 days, the levels of *Bifidobacterium* in Sal groups (0.43–2.40%) were higher than those in the Con (0.04%) and Mod (0.20%) groups, among which the most prominent increase in *Bifidobacterium* occurred in the Sal50 and Sal100 groups.

**Figure 6:**
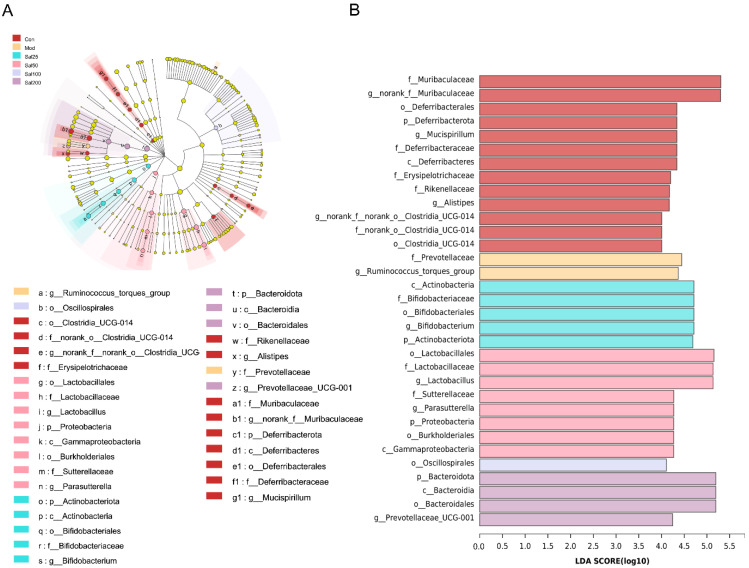
An linear discriminant analysis (LDA) effect size (LEfSe) analysis and LDA score for taxa differing between six groups. (**A**) The taxonomic cladogram obtained from the LEfSe analysis of gut microbiota. (**B**) The LDA score obtained from LEfSe analysis of gut microbiota in different groups. LDA score was set at 4.

To further identify the specific differences of gut microbiota from phylum to genus under the regulation of salidroside, linear discriminant analysis (LDA) effect size (LEfSe) was carried out (Figure 6). Differentially abundant fecal bacterial taxa in antibiotic-treated mice in response to salidroside were identified by LEfSe analysis. It was noteworthy that the intake of low-dose salidroside caused an enrichment of well-known probiotics *Lactobacillus* and *Bifidobacterium*. The family of *Bifidobacteriaceae*, genus of *Bifidobacterium* and phylum of *Actinobacteriota* in Sal25 group, and order of *Lactobacillales*, family of *Lactobacillaceae*, and genus of *Lactobacillus* in Sal50 group, were enriched. *Bacteroidota* in the Sal200 group, *Lactobacillus* in the Sal50 group, and *Bifidobacterium* in the Sal25 group were predicted to be the main dominant bacteria and biomarkers for regulating the disturbed gut microbiota.

#### 3.2.3. Salidroside Improved the SCFA Production

**Figure 7:**
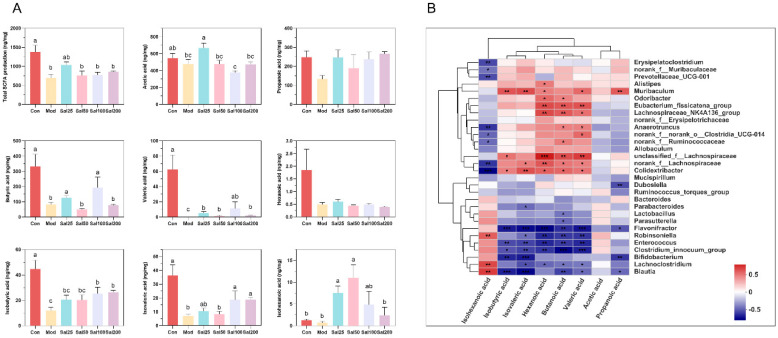
The effects of salidroside on short-chain fatty acid (SCFA) production. (**A**) The contents of SCFAs. The values are displayed as the mean ± SEM. Small letters show significant differences between groups (*p* < 0.05). (**B**) Heatmap of correlation analysis between gut microbiota and SCFAs (* *p* < 0.05, ** *p* < 0.01, *** *p* < 0.001).

Gut microbiota can metabolize carbohydrates and produce SCFAs with attractive benefits. SCFAs, mainly comprising acetic acid, propionic acid, butyric acid, hexanoic acid, isobutyric acid, isovaleric acid, and isohexanoic acid, serve as important molecular signals between the microbiota and host and also as metabolic substrates regulating host cellular metabolism [28]. They are important fuels for intestinal epithelial cells and regulate their functions such as strengthening the gut barrier functions [29]. Considering the glycoside structure of salidroside, the SCFAs produced by gut microbiota were further analyzed after treatment with salidroside. The contents of the detected SCFAs, including acetic acid, butyric acid, valeric acid, isobutyric acid, isovaleric acid, and isohexanoic acid, all increased significantly in some of the Sal groups on the 7th day compared with the Mod group (Figure 7). The contents of acetic acid in the Sal25 group, butyric acid in the Sal25 and Sal100 groups, valeric acid in the Sal100 group, and isovaleric acid in the Sal25, Sal100, and Sal200 groups nearly recovered to the relevant levels of the Con group. It is worth mentioning that the contents of isohexanoic acid in Sal25 and Sal50 even exceeded that in the Con group. The gut microbiota correlated with SCFA production was analyzed at the genus level, and a heatmap of spearman correlation analysis was generated (Figure 7). It showed that *Lachnospiraceae_NK4A136_group*, *Odoribacter*, *Anaerotruncus*, *norank_f_Ruminococcaceae*, *unclassified_f_Lachnospiraceae*, *norank_f_Lachnospiraceae*, and *Eubacterium_fissicatena_group* were positively correlated with the production of butyric acid, whereas *Blautia*, *Enterococcus*, and *Flavonifractor* were negatively correlated with the production of butyric acid.

## 4. Discussion

Gut microbiota is considered a virtual organ of the human body, playing a crucial role in human health and disease [3]. However, the abuse of broad-spectrum antibiotics can cause dysbiosis of gut microbiota, leading to secondary infections, antibiotic-associated diarrhea, and a variety of other diseases [2]. From another point of view, gut microbiota dysbiosis is also the character of intestinal diseases, and improving gut microbiota may be associated with disease recovery [26]. Many phenols with little bioavailability can evade stomach and small intestine digestion and reach the colon. There they encounter the gut microbes, resulting in a two-way interaction in which phenols promote the proliferation of helpful bacteria through fermentation and improve gut microbial diversity, and the intestinal microbes metabolize the phenols to produce beneficial metabolites, most notably SCFAs [30]. Tea, pomegranate extract and many other phenolic-containing foods have shown “prebiotic-like” effects, such as inducing regulation of the gut microbiota and resulting in ultimate health benefits [26,30,31]. Thus, phenols meet the novel definition states that “a prebiotic is a non-digestible compound that through its metabolization by microorganisms in the gut, modulates the composition and/or activity of the gut microbiota, thus conferring a beneficial physiological effect on the host” [32]. And that is why the recently created concept of “three P” s for gut health includes probiotics, prebiotics, and phenols [33]. In this work, salidroside, a phenolic compound, was first found to have similar functions of prebiotics and modulate gut microbiota disturbance and its metabolites, namely, enriching probiotics and increasing the SCFA production.

Salidroside contributed to the recovery of the intestinal damage and inflammation induced by the overuse of ceftriaxone sodium at 200–800 mg/kg/day. Overusing antibiotic ceftriaxone sodium in mice led to intestinal damage, which was manifested as a loss of goblet cells in the ileum, colonic mucosal edema, and cecum edema and darkness. Moreover, the spleen index of the mice was decreased, and the levels of ALT and AST as well as inflammation factors TNFα and IL-6 in serum were all significantly increased, suggesting that excessive antibiotics could induce systemic inflammation. Consistent with this, some researchers have found the same results in experiments, and considered this may be associated with alteration of occludin in intestinal [34]. After a 7-day administration of salidroside, the damage to the intestinal structure was improved, whereas the Mod group still had ileal villi breakage and colon inflammatory infiltration. Histological analysis further showed obvious attenuation of inflammatory cell infiltration and mucosal damage in the illum. Salidroside relieved the immunosuppression of ceftriaxone sodium on the spleen as preliminarily exemplified by the increase of spleen indexes in all Sal groups. Furthermore, essential inflammation factors were all decreased compared to the Mod group, indicating the reduction of systemic inflammation. The repair of intestinal tissue reduced the risk of transfer of toxins produced by some bacteria, which was confirmed by a decrease of inflammatory factors in serum, suggesting that salidroside enhances systemic immunity [19,34]. The main factors responsible for the beneficial effects and the relationship between gut microbiota and intestinal phenotype were still not clear, which would require further investigation in the future work.

Salidroside played an important role in regulating the community richness and diversity. Gut microbiota in the mice treated with 400 mg/kg/day ceftriaxone sodium was found disturbed, with the community richness and diversity severely reduced and the community structure highly destroyed, which has been found by previous studies [34,35]. Microbial dysbiosis may be associated with adverse effects such as mucosal leakage, intestinal and systemic inflammation [3]. However, salidroside promoted the recovery of gut microbiota richness, diversity, and community structure in mice. High-dose salidroside helped mice restore the richness and diversity of gut microbiota to the normal level. Notably, high diversity and richness of gut microbiota are not indicators of healthy microbiota, since the flora richness and diversity are influenced by a variety of factors. For example, a prolonged transit time of food may result in an increased richness but not equal to healthy gut microbiota [3]. PCoA showed that the community structure of the high-dose salidroside groups tended to be close to the Con group, indicating their similarity in community structure.

Salidroside exhibited a significant restorative effect at the phylum level of the gut microbiota disturbance caused by ceftriaxone sodium. After ceftriaxone sodium treatment, the abundance of *Firmicutes* exceeded that of *Bacteroidota* at the phylum level, and the phyla of *Verrucomicrobiota* and *Actinobacteria* were barely detectable owing to the indiscriminate killing effects of antibiotics on the flora. By contrast, the abundance of *Parabacteroides* was increased due to its wide spectrum of resistance to various β-lactam antibiotics, which was also found in human who taken with ceftriaxone [22,36]. The decreased abundance of *Actinobacteria* and increased abundance of *Parabacteroides* were also observed in the ceftriaxone-treated mice in the previous reports [35]. Antibiotic-induced microbiome depletion may alter metabolic homeostasis by affecting gut signaling and colonic metabolism [37]. After salidroside treatment, the abundance of *Bacteroidota* exceeded that of *Firmicutes*, which was different from that of ceftriaxone sodium treatment. It was reported that the *Firmicutes*/*Bacteroidetes* ratio was higher in healthy vegetarians than that in non-vegetarians [38]. However, it was also reported that the ratio was also higher in obese animals and humans, since *Firmicutes* were more efficient in extracting energy from food and thus promoted more efficient calorie absorption and weight gain [39,40]. In addition, the undetectable phylum *Actinobacteria* and *Verrucomicrobiota* after antibiotic treatment were increased after the administration of salidroside, among which *Actinobacteria* was considered to have possible therapeutic use for gastrointestinal and systemic diseases [41]. *Verrucomicrobia* and *Actinobacteria* were found to act as gut microbiota biomarkers on salidroside treatment to alleviate furan-induced liver injury in a mouse model [17].

Importantly, salidroside modulated the gut microbiota at the genus level, namely, promoting the proliferation of some beneficial bacteria and inhibiting the growth of some disease-related bacteria, consistent with the findings in other disease models with salidroside-treatment [17,18,19]. In the presence of 400 mg/kg/day ceftriaxone sodium, the genera of *norank_f_Muribaculacea* and *Allobaculum* almost undetectable. Among them, *Allobaculum* was reported to be positively correlated with the production of SCFAs [42]. By contrast, the genus of *Enterococcus* survived and its abundance was increased, which might be related to their high resistance toward β-lactam antibiotics [43]. The ceftriaxone-resistant *Enterococcus,* a high risk among patients, has become a major global public health threat [23]. Nevertheless, the intake of salidroside reversed the above situation. Salidroside can promote the growth of certain beneficial genera such as *Bacteroides*, *Actinobacteria*, *Parabacteroides*, *Dubosiella, Lactobacillus*, and *Bifidobacterium.* The genus of *Bacteroides* plays an important role beneficial to human metabolism, and its abundance increase with weight loss in obese patients and the lean population [44]. *Parabacteroides* exert protective effects against multiple sclerosis, type II diabetes, colorectal cancer, and inflammatory bowel disease, whereas some studies showed the evidence of potential pathogenic effects of this genus [36]. The proliferation of *Dubosiella* contributes to alleviating salt-sensitive hypertension [45]. The genera of *Lactobacillus* and *Bifidobacterium* are well known probiotics with a range of health benefits. Owing to the excellent promotion of these two classic probiotics, salidroside was confirmed to have similar prebiotic functions. On the other hand, salidroside inhibited the proliferation of some disease-related genera, such as *norank_f_Muribaculaceae*, *Helicobacter*, and *Ruminococcus_torques_group*. The gastrointestinal infections associated with *Helicobacter pylori* occur commonly worldwide, which are mainly related to the occurrence of peptic ulcers, gastritis, and gastric cancer [46]. The supplement of *Ruminococcus_torques_group* has been reported to exacerbate the symptoms of the complex neurodegenerative disorder, amyotrophic lateral sclerosis, leading to a degeneration of motor neurons [47]. In patients with age-related macular degeneration and Hashimoto’s thyroiditis, the abundance of the *Ruminococcus_torques_group* is significantly increased [48,49]. The inhibition of salidroside on the growth of disease-related microbiota might contribute to restoring healthy gut microbiota.

It is worth noting that the low-dose salidroside, including the amount used in the Sal25 and Sal50 groups, has different effects on gut microbiota compared to the high-dose salidroside groups. The Sal25 and Sal50 groups showed lower diversity and richness in gut microbiota, based on the slightly lower Chao and Shannon indexes. Additionally, the composition of Sal50 on the 7th and 14th days and Sal25 on the 7th day were significantly distinct from other groups, based on the results of PCoA. According to the Veen plot, the unique OTUs of Sal25 and Sal50 were increased from day 7 to day 14. The promotion effects of the low-dose salidroside on the beneficial bacteria such as the phyla of *Actinobacteria* and *Verrucomicrobiota* and the genera of *Parabacteroides*, *Dubosiella*, *Lactobacillus,* and *Bifidobacterium,* were more prominent than the high-dose salidroside. Even more importantly, by LEfSe analysis, *Lactobacillus* and *Bifidobacterium* in low-dose salidroside groups were finally confirmed to be the domain bacteria and biomarkers that contributed to gut microbiota rebuilding. The inhibition of low-dose salidroside on disease-associated genus *norank_f_Muribaculaceae* was also more obvious than high-dose salidroside.

Additionally, the intake of salidroside completely reversed the attenuation of SCFAs in antibiotic-treated mice. After treatment with ceftriaxone sodium, the SCFAs in mice were almost undetectable (Figure S4). This phenomenon has also been reported in Lincomycin-treated mice [11]. However, with the treatment of salidroside for 7 days, a significant increase occurred in the contents of SCFAs, including acetic acid, butyric acid, valeric acid, isobutyric acid, isovaleric acid, and isohexanoic acid. As the main source of energy for colon cells, butyric acid plays an important role in maintaining the integrity of the intestinal barrier, improving colonic defense barrier function, modulating immune and inflammatory responses, preventing colon carcinogenesis, promoting satiety, and maintaining an anaerobic environment in the intestinal lumen [50,51]. It was reported that butyric acid contributes to restoring intestinal damage and inflammation induced by ceftriaxone, mainly through direct inhibition of histone deacetylase and/or interaction with its receptors GPR41 and GPR43 [34]. The predicted butyric acid-producing bacteria in this study, such as *Lachnospiraceae_NK4A136_group*, *Odoribacter*, *Anaerotruncus*, *norank_f_Ruminococcaceae*, *unclassified_f_Lachnospiraceae*, *norank_f_Lachnospiraceae*, and *Eubacterium_fissicatena_group*, had also been reported to produce butyrate acid in the previous literatures [28,51,52,53]. The restoration of SCFA contents and gut microbiota could improve intestinal barrier functions, help resist the inflammatory response, and contribute to the repair of antibiotic-induced intestinal damage.

## 5. Conclusions

Salidroside was for the first time found to alleviate gut microbiota disturbance induced by excessive antibiotics. Salidroside diminished levels of inflammatory cytokines and promoted intestinal damage repair. It increased the abundances of beneficial species and reduced the abundances of disease-related species*,* thus reshaping the gut microbiota. Low-dose salidroside can play a superiorly beneficial role in a short time. Moreover, the contents of SCFAs were significantly increased after the intake of salidroside, which might contribute to the recovery of intestinal function. All of these results suggested salidroside had similar functions of prebiotics and it would be a promising dietary supplement for improving antibiotic-induced gut microbiota dysbiosis and related disease. The mechanism of the promotion or inhibition effects of the salidroside on intestinal flora, as well as the relationship between the alteration of gut microbiota and the recovery of gut damage need to be further investigated.

## Data Availability

Data is contained within the article and supplementary material.

## Supplementary Materials

The following supporting information can be downloaded at: https://www.mdpi.com/article/10.3390/foods11193073/s1, Figure S1: The effects of excessive ceftriaxone sodium on mouse weight (A), spleen index (B), and serum concentrations of ALT (C), AST (D), IL-6 (E), and TNFα (F); Figure S2: The effects of excessive ceftriaxone sodium on mice intestinal damage; Figure S3: The effects of excessive ceftriaxone sodium on gut microbiota; Figure S4: The effects of excessive ceftriaxone sodium on SCFA production.
