# Supplementary material for: Salidroside Improves Antibiotic-Induced Gut Microbiota Disturbance and Low Levels of Short-Chain Fatty Acids in Mice
Source: Foods. 2022 Oct 3;11(19):3073. doi: 10.3390/foods11193073 (PMC9564336; doi:10.3390/foods11193073)
Supplement: Supplementary file 1 [file foods-11-03073-s001.zip › foods-1906732-supplementary.pdf]

Supplementary Materials

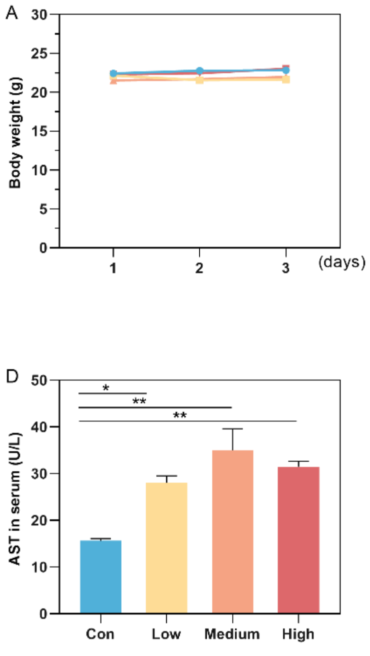

Figure S1. The effects of excessive ceftriaxone sodium on mouse weight (**A**), spleen index (**B**), and serum concentrations of ALT (**C**), AST (**D**), IL-6 (**E**), and TNF*α* (**F**). The values are displayed as mean ± SEM. *$p < 0.05$, **$p < 0.01$, ***$p < 0.001$.

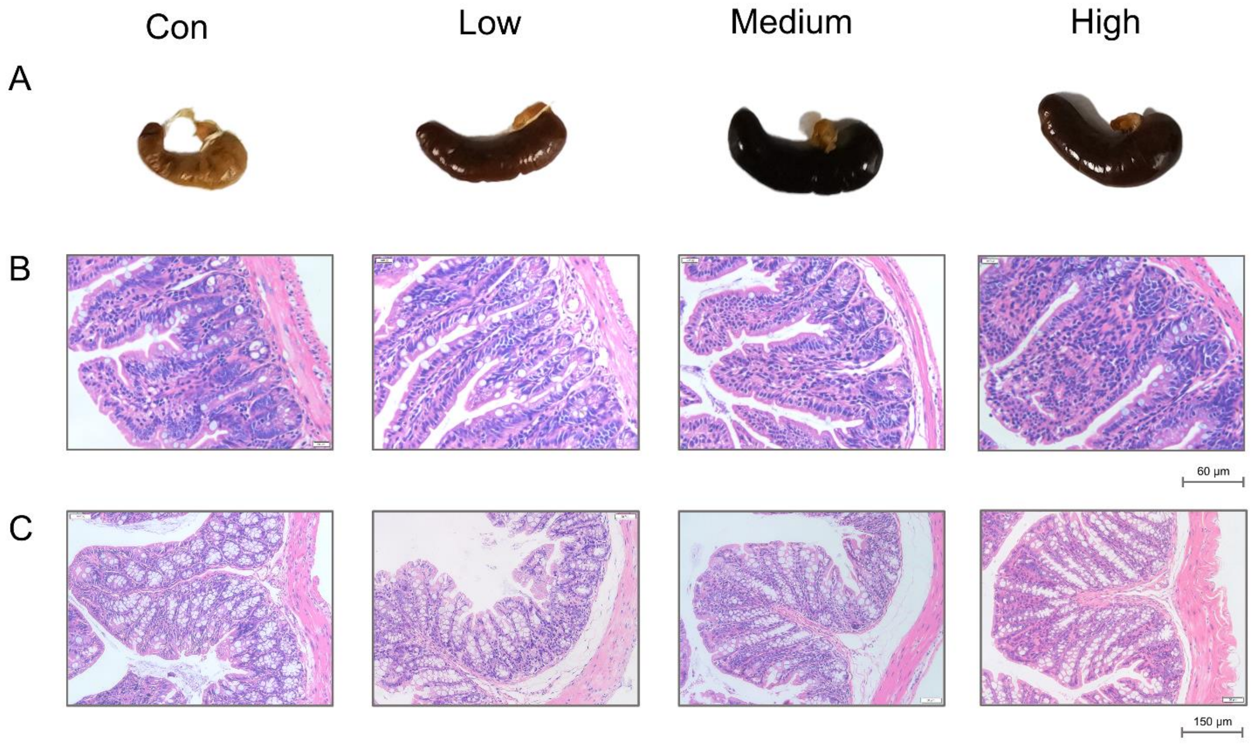

Figure S2. The effects of excessive ceftriaxone sodium on mice intestinal damage. (**A**) Cecum tissues. (**B**) Ileum tissues. (**C**) Colon tissues.

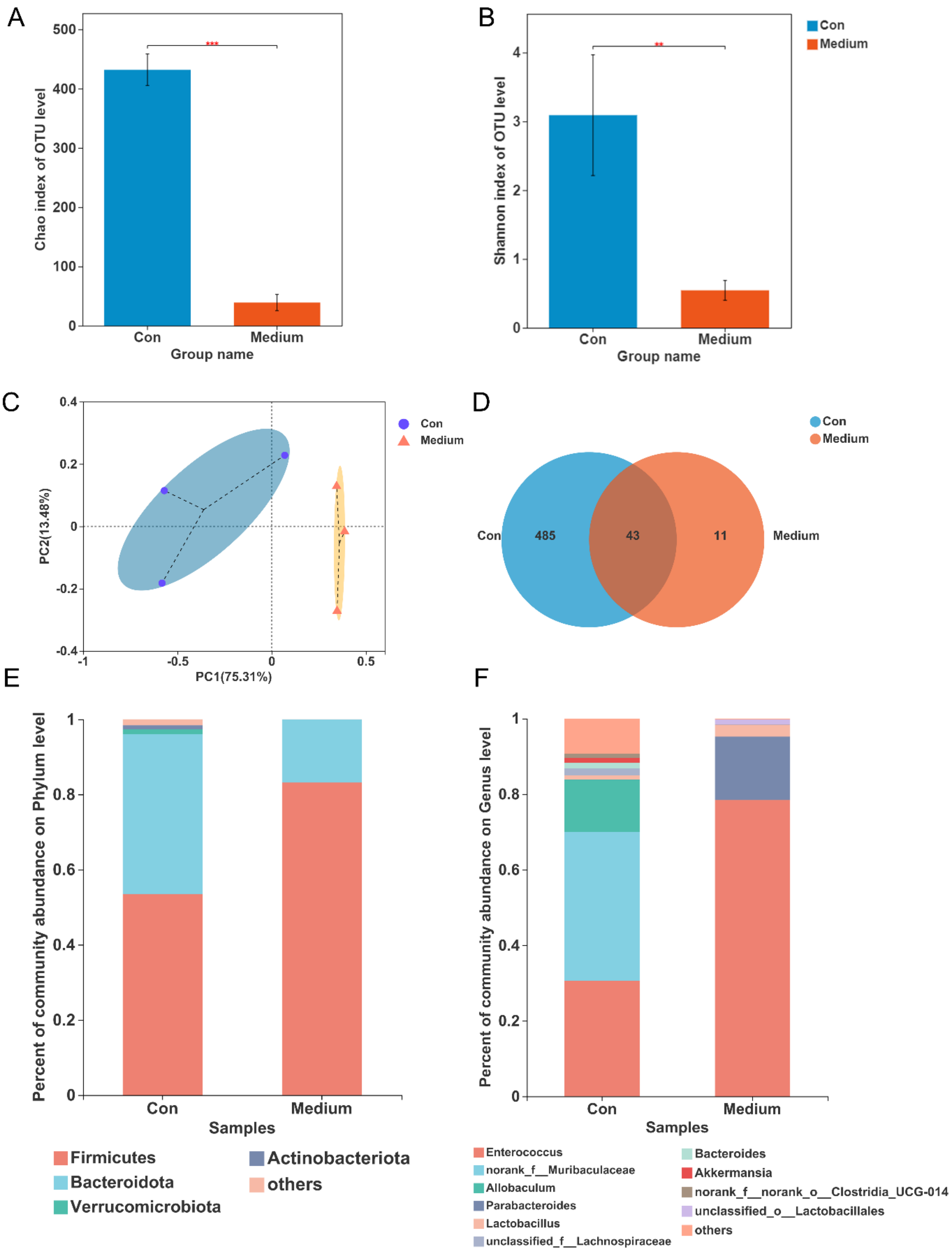

Figure S3. The effects of excessive ceftriaxone sodium on gut microbiota. (**A**) Chao index. (**B**) Shannon index. (**C**) PCoA analysis. (**D**) Veen analysis. (**E, F**) The composition at the phylum and genus level, respectively. $^{*}p < 0.05$, $^{**}p < 0.01$, $^{***}p < 0.001$.

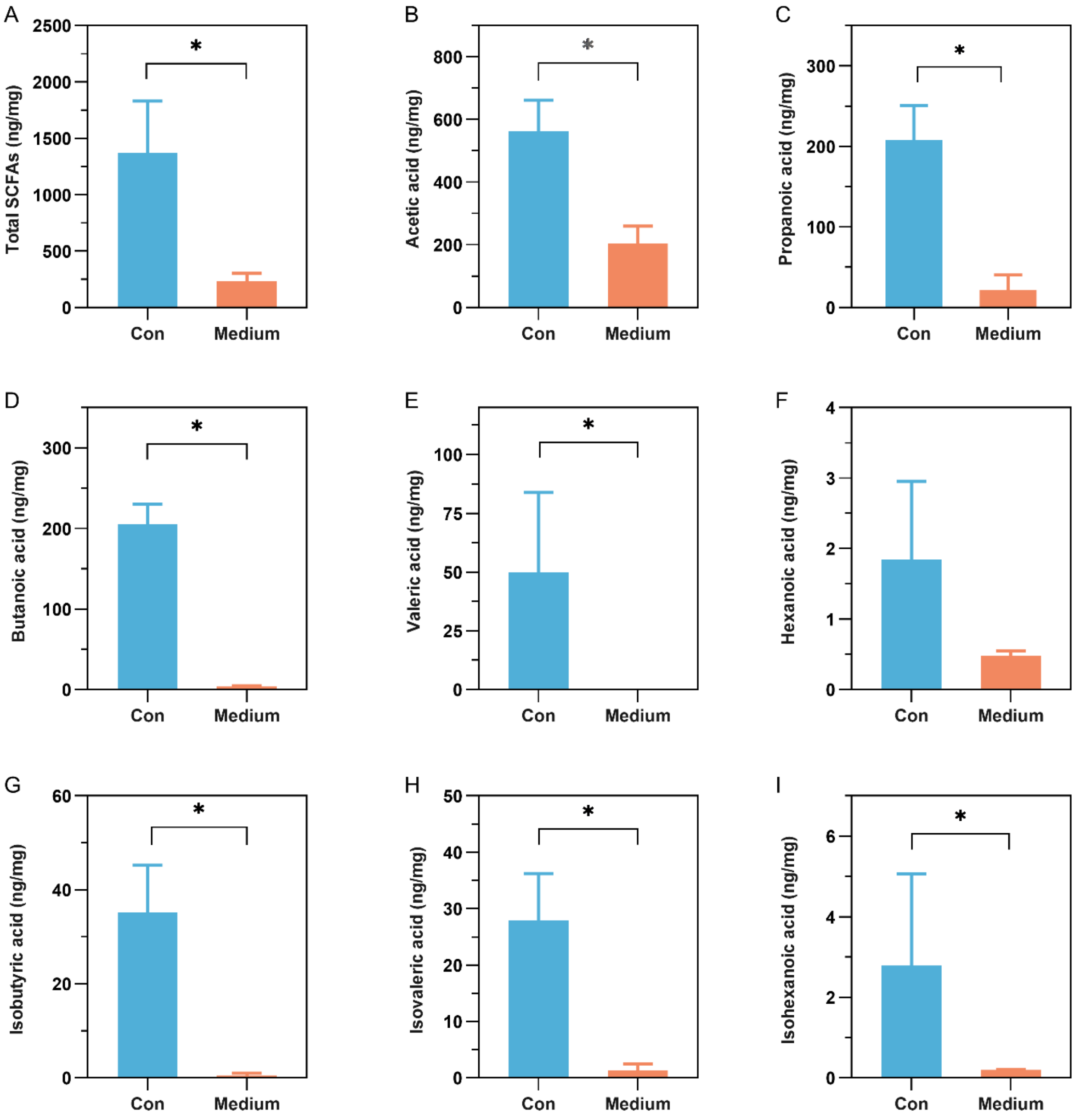

Figure S4. The effects of excessive ceftriaxone sodium on SCFA production. The values are displayed as the mean ± SEM. *$p < 0.05$.
